# Epigenetic field for cancerization: its cause and clinical implications

**DOI:** 10.1186/1753-6561-7-S2-K22

**Published:** 2013-04-04

**Authors:** Toshikazu Ushijima

**Affiliations:** 1 Division of Epigenomics, National Cancer Center Research Institute, Japan

## 

Epigenetic alterations are present not only in cancer cells but also in non-cancerous tissues. Accumulation levels of aberrant DNA methylation in non-cancerous tissues can correlate with risk of cancer development, especially in chronic inflammation-associated cancers [1-3]. The close correlation in non-cancerous tissues was prominent for epigenetic alterations, compared with genetic alterations, and formed a concept of "epigenetic field for cancerization (epigenetic field defect)". In gastric cancers, close correlation between methylation levels and cancer risk has been demonstrated [4].

As mechanisms for methylation induction in the stomach, infection by *Helicobacter pylori* (*H. pylori*), the major cause of gastric cancers, was implicated in humans [5], and was demonstrated in *Mongolian gerbils *[6]. Especially, a critical role of inflammation triggered by *H. pylori* infection, not by high concentrations of ethanol or salt, was demonstrated, suggesting the importance of specific chronic inflammation [7]. Gene expression analysis showed that expression levels of *Il1b*, *Nos*, and *Tnf* were well correlated with methylation levels induced.

To dissect molecular mechanisms for induction of epigenetic alterations, a mouse colitis model induced by dextran sulfate sodium (DSS) was used. First, we isolated genes methylated in colon tumors induced by DSS and azoxymethane, and showed that these genes were methylated in non-cancerous colonic mucosae, forming an epigenetic field. Aberrant methylation was induced even in SCID mice, which lack functional T- and B-lymphocytes, and it was shown that lymphocytes are not essential in methylation induction [8]. By chromatin-immunoprecipitation-on-Chip analysis of H3K27me3, aberrant H3K27me3 was shown to be induced by colitis, and can be carried into cancer tissues and function as a premark for induction of aberrant DNA methylation [9].

One of the major translations of the epigenetic field for cancerization is its use as a cancer risk marker. By searching for CpG islands differentially methylated in gastric mucosae of gastric cancer patients and healthy volunteers, both of which had past infection by *H. pylori*, we were able to isolate and validate seven differentially methylated CpG islands. The new markers had large areas under the receiver-operating characteristic curves (0.78-0.84) and high odds ratios (12.7-36.0) even among individuals with past *H. pylori* infection, compared with two currently available markers (0.60-0.65, 5.0-5.7) [10]. We are currently conducting a prospective study to predict patients who suffer from metachronous gastric cancers among gastric cancer patients treated by endoscopic submucosal dissection.

Another translation is prevention of cancers. Several studies involving viral oncogenes and chemical carcinogens showed that epigenetic cancer prevention is possible, but there have been no studies for the usefulness of epigenetic cancer prevention in chronic inflammation-associated cancers. We administered 5-aza-2'-deoxycitidine (5-aza-dC) to Mongolian gerbils infected with *H. pylori* after administration of *N*-methyl-*N*-nitrosourea. It was shown that the incidence of gastric cancers was suppressed almost to half of that in gerbils without 5-aza-dC [Niwa, submitted].

These findings vividly show that the epigenetic field defect has its unique characteristics, such as ease of measurement and reversibility, and harbors a rich chance of clinical translations.

## Competing interests

There are no competing interests in this presentation.
